# A Novel Toolkit for Characterizing the Mechanical and Electrical Properties of Engineered Neural Tissues

**DOI:** 10.3390/bios9020051

**Published:** 2019-04-01

**Authors:** Meghan Robinson, Karolina Papera Valente, Stephanie M. Willerth

**Affiliations:** 1 Biomedical Engineering Program, University of Victoria, Victoria, B.C. V8W 2Y2, Canada; meghanro@uvic.ca; 2 Department of Mechanical Engineering, University of Victoria, Victoria, B.C. V8W 2Y2, Canada; kvalente@uvic.ca; 3 Division of Medical Sciences, University of Victoria, Victoria, B.C. V8W 2Y2, Canada; 4 Centre for Biomedical Research, University of Victoria, Victoria, B.C. V8W 2Y2, Canada

**Keywords:** tissue engineering, rheology, electrophysiology, biomedical devices, elastic modulus

## Abstract

We have designed and validated a set of robust and non-toxic protocols for directly evaluating the properties of engineered neural tissue. These protocols characterize the mechanical properties of engineered neural tissues and measure their electrophysical activity. The protocols obtain elastic moduli of very soft fibrin hydrogel scaffolds and voltage readings from motor neuron cultures. Neurons require soft substrates to differentiate and mature, however measuring the elastic moduli of soft substrates remains difficult to accurately measure using standard protocols such as atomic force microscopy or shear rheology. Here we validate a direct method for acquiring elastic modulus of fibrin using a modified Hertz model for thin films. In this method, spherical indenters are positioned on top of the fibrin samples, generating an indentation depth that is then correlated with elastic modulus. Neurons function by transmitting electrical signals to one another and being able to assess the development of electrical signaling serves is an important verification step when engineering neural tissues. We then validated a protocol wherein the electrical activity of motor neural cultures is measured directly by a voltage sensitive dye and a microplate reader without causing damage to the cells. These protocols provide a non-destructive method for characterizing the mechanical and electrical properties of living spinal cord tissues using novel biosensing methods.

## 1. Introduction

Neural tissue engineering generates substitutes for damaged or diseased regions of the central nervous system by combining biomaterials, cells and drug delivery systems [1,2]. Biomaterials are natural or synthetic cross-linked materials that mimic the microenvironment of the extracellular matrix, and they can be seeded with stem cells to form neural tissues [3]. Stem cells possess the ability to mature into multiple distinct cell types, making them a powerful tool for tissue engineering applications [4]. It is important to be able to confirm that these engineered tissue constructs possess the mechanical and electrical signaling associated with native neural tissue. This paper details (1) a novel method for measuring the mechanical properties of such hydrogels and (2) a non-destructive method for examining the electrophysiological activity of these cells.

Stem cells can sense and respond to the mechanical stiffness within their microenvironment by way of surface integrins, which are intricately connected to their cytoskeletal structure, causing inhibition or promotion of growth, and alterations within the shape of the nucleus leading to chromatin remodeling and changes in gene expression [5,6]. Thus, the mechanical stiffness, or elastic modulus, of engineered tissue scaffolds must closely reflect that of the natural tissue for tissue regeneration to occur. Indeed, neurons prefer softer scaffolds below 1 kPa, and neural regeneration requires scaffold softness to be between 0.1–0.5 kPa, whereas slightly stiffer scaffolds of ~3.5 kPa will instead induce neural precursor cell proliferation [7], and those between 1 and 10 kPa will induce astrocytic fates from neural progenitor populations [8,9]. Current standards for measuring the elastic modulus of soft scaffolds like hydrogels are limited to indirect relationships between elastic modulus and other measurements, such as shear stress in the case of rheology or tip indentation in the case of atomic force microscopy (AFM) [5,10,11,12]. These methods use mathematical modeling to relate experimentally determined displacement and load measurements to strain and stress values in order to calculate elastic modulus, and this requires assumptions of ideal deformations. For soft water-based materials like hydrogels, their low viscosity and slippery behavior can violate these ideal conditions leading to misinterpretation of elastic modulus [13,14]. There is currently a lack of suitable tools for reliably characterizing the ultra-soft extracellular matrix required for regenerating neural tissues such as those found in the spinal cord, as illustrated by the large disparity in published results of neural tissue biomechanics, both in experimental findings and theoretical models [15].

Here we describe a protocol for determining elastic modulus of soft hydrogels using a modified version of the Hertz model. Our method measures elastic moduli based on the indentation depth created by a spherical indenter on the hydrogel surface. By labeling the hydrogel samples with fluorescent dyes, the indentation depth created by the sphere can be easily determined using a fluorescence microscope. This method avoids the risk of misinterpretation of data present with indirect methods such as rheology and AFM by directly correlating the indentation depth and hydrogel thickness with elastic modulus.

Neurons transmit information by sending electrical signals, thus confirming that engineered tissues can replicate these signals serves as a critical validation step. This can be accomplished by measuring cell membrane potential. As neurons mature, their resting membrane potential hyperpolarizes until it reaches −70 mV. Immature neurons can be identified by having a more depolarized resting membrane potential than mature neurons. Moreover, neurons are considered functional when they can respond to stimulation by firing action potentials: all or nothing rapid membrane potential spikes of +40 mV amplitude, triggered when a cell’s membrane potential reaches a threshold of −55mV. These electrophysical fluctuations can be monitored through a process called patch clamping, in which neurons are punctured with electrodes in order to measure changes in membrane potential associated with the opening and closing of ion channels distributed along the cell’s membrane [16]. However, this process destroys the cells being analyzed, is labor intensive, requires highly specialized training and is restricted to the analysis of single cells. Another common and less damaging method is to indirectly determine action potential activity by measuring intracellular calcium fluctuations. This technique falsely assumes that action potentials are the only reason for calcium level fluctuations within the cells. This assumption becomes particularly risky for neurons, as calcium influx mediates many other intracellular functions, and becomes dysregulated in neurodegenerative diseases like Alzheimer’s Disease, Parkinson’s Disease, and Amyotrophic Lateral Dystrophy [17,18]. In addition to being an indirect reporter of electrical activity, intracellular calcium level fluctuations are significantly slower than action potentials making them incapable of reporting membrane potential fluctuations in real time [19].

Here we describe a system incorporating the use the voltage sensitive fluorescent dye FLIPR Blue (Fluorescence Imaging Plate Reader Blue dye) as a direct and robust tool for reporting the electrical activity of cell cultures [20,21,22]. FLIPR Blue, a cell-permeant dye with a high signal-to-noise ratio, is internalized upon membrane depolarization, where it binds to intracellular hydrophobic sites and emits fluorescence. Likewise, membrane hyperpolarization reverses internalization of the dye and fluorescent emission is decreased, enabling detection of electrophysical activity in real time. Moreover, this dye can be applied to cell cultures without affecting their viability, providing a key advantage over patch clamping.

Both protocols introduce advantages in terms of cell viability and accuracy acquired through direct measurements. While these methods have been described previously this is the first instance that we know of where they have been optimized and validated for measuring the ultra-soft fibrin hydrogels commonly used in spinal cord regeneration procedures, and for detecting electrophysical functionality in the resulting regenerated tissues without loss of cell viability. Used together these tools provide both electrophysical and mechanical analysis, both of which are critical for regeneration of functional spinal cord tissue.

## 2. Materials and Methods

### 2.1. Materials

Fibrin (341578), thrombin (605190) and calcium chloride (102391) were purchased from Millipore Sigma (Burlington, MA, USA), fluorescein sodium salt (46940) from Sigma Aldrich (St. Louis, MO, USA), and fluorescent polystyrene particles from Magsphere Inc. (Pasadena, CA, USA). Spherical indenters (1/32″ zirconium dioxide (ZrO_2_) and 1/8″ tungsten carbide (WC)) were purchased from BearingsCanada (Woodbridge, ON, Canada). Human motor neurons were purchased from BrainXell (Madison, WI, USA) (BX-0100), along with Seeding Supplement and Day 4 Supplement. Human induced pluripotent stem cells (hiPSCs, cell line 1-DL-01) were purchased from WiCell Research Institute (Madison, WI, USA). Dulbecco’s Modified Eagle Medium/F12 (DMEM/F12) Medium (11330-032), Neurobasal Medium (21103-049), B27 Supplement (17504-044), N2 Supplement (17502-048), GlutaMAX (35050-061), and Geltrex (A1413201) were purchased from Life Technologies (Carlsbad, CA, USA). Brain derived neurotrophic factor (BDNF, 450-02), Glial derived neurotrophic factor (GDNF, 450-10) and Transforming growth factor beta 1 (TGF-β1, 100-21C) were purchased from PeproTech (Rocky Hill, NJ, USA). Poly-L-Ornithine (PLO, P4957), Laminin from Engelbreth-Holm-Swarm murine sarcoma basement membrane (L2020), potassium chloride (KCl, P9541) and acetylcholine chloride (A2661) were purchased from Sigma Aldrich. FLIPR Membrane Potential Assay Kit Blue (R8042) was purchased from Molecular Devices (San Hose, CA, USA). TeSR-E8 Medium (05990), ReLeSR (05872), Gentle Cell Dissociation Reagent (07174), StemDiff Neural Induction Medium (NIM, 05835), StemDiff Neural Rosette Selection Reagent (05832) Aggrewell 800 Starter Kit (34850), 37 µm reversible strainers (27250), cell lifters (38067) and Stemdiff Neural Progenitor Media (05833) from Stemcell Technologies (Vancouver, BC, Canada). Phosphate buffered saline (PBS, 10-010-049) was purchased from Fisher Scientific (Hampton, NH, USA). Calcium chloride (CA71006-874) was purchased from Anachemia Chemicals, Inc. (New York, NY, USA). Tris Base (8980-1) was purchased from Caledon Laboratories Ltd. (Georgetown, ON, Canada).

### 2.2. Cell Culture

Human motor neurons were cultured as per the supplier’s instructions (BrainXell document v4.2). In short, seeding medium was prepared by mixing DMEM/F12 Media (0.5X), Neurobasal Media (0.5X), N2 supplement (1X), B27 supplement (1X), GlutaMAX (0.5 mM), and Seeding Supplement (1X). Cells were seeded at 20,000 cells/well, 50,000 cells/well, 100,000 cells/well and 200,000 cells/well into a 96-well plate coated with PLO/laminin. On day 1, media was replaced with the additional elements of BDNF (10 ng/mL), GDNF (10 ng/mL), TGFβ (1 ng/mL), and Geltrex (15 µg/mL). On day 4, media was replaced with Seeding Supplement replaced by Day 4 Supplement (1X) and without the addition of Geltrex. On day 7 cells were analyzed.

hiPSCs were cultured in xeno-free conditions on vitronectin substrates in TeSR-E8 media, which media changes every day until they reached 90% confluence. Neural progenitors were derived by transferring hiPSCs to AggreWell800 plates for five days in Neural Induction Media to create neural aggregates. Neural aggregates were then plated on poly-L-ornithine (PLO)/laminin coated plates and cultured in NIM for a further seven days. Neural rosettes appeared at this time and were selected by incubation at 37 °C for 45 min in Neural Rosette Selection Reagent, followed by pipetting. Rosettes were then transferred to a fresh PLO/laminin coated plate and cultured in NIM until confluent. Once confluent, cells were detached from the plate by incubating in Gentle Cell Dissociator Reagent for 3 min at room temperature, rinsing with DMEM and scraping with a cell lifter into Stemdiff Neural Progenitor Media. Cells were then pipetted into single cells using a 1000 µL pipette and transferred onto a fresh PLO/laminin coated plate, and cultured in Stemdiff Neural Progenitor Media until 100% confluent, with media changes every day.

### 2.3. Preparation of Fibrin Hydrogels

Fibrin hydrogels were prepared according to the protocol “Preparation of 3D Fibrin Scaffolds for Stem Cell Culture Applications” by Willerth et al. [23]. A total of 1 mL of fluorescein was added to scaffolds and incubated overnight before measurements were made.

### 2.4. FLIPR Blue Prep and Imaging

Cells were incubated in FLIPR Blue dye as per the manufacturer’s instructions, by adding precisely a 1:1 ratio of dye to cell culture media to the cells and incubating for 30 min at 37 °C in the dark. The plates were then read by a microplate reader set to excite fluorescence at 530 nm and read fluorescent emission at 565 nm, taking 25 reads from each well in 5 × 5 grid.

Cells were visualized with a Leica DMI 3000B microscope equipped with an XCite Series 120Q fluorescent light source and QImaging RETIGA 2000R camera at 100 × magnification. Images were captured using QCapture Software 2.9.12. Fluorescently labeled cells were visualized with a green filter for green fluorescent protein. To better visualize the signal from the FLIPR Blue dye, gain was set to its maximum setting and exposure to its minimum at around 150 ms/frame. For time-lapse image stacks images were taken 1/500 ms.

### 2.5. Statistics

Statistical significance was performed by one-way student *t*-test assuming two sample equal variance for the mature versus immature neurons comparison, and by one-way paired student *t*-test for the stimulated versus at rest comparison.

### 2.6. Design and Validation of an Indentation Protocol for Measurement of Elastic Modulus of Hydrogels

Elastic modulus of each hydrogel sample was determined by the indentation method [24,25,26]. Fibrin samples of 500 uL volume each with concentrations of 10, 20 and 30 mg/mL were prepared in a 24-well plate in triplicates using 40 U/mL thrombin and 50 mM calcium chloride as a crosslinker. After crosslinking of fibrin samples, 2 mL of a 50 μM fluorescein sodium salt solution was added to each sample in order to fluorescently stain the hydrogel. This staining allowed measurement of hydrogel thickness and indentation. After overnight incubation with fluorescein solution at 37 °C, hydrogel samples were washed three times with 1 × PBS in order to remove excess of fluorescein dye. In order to clearly define the hydrogel surface, 2 mL of a water solution containing fluorescent polystyrene particles was deposited on the surface of the fibrin samples. The well was then moved to the sample stage of the Laser Scanning Microscope (LSM 880 Zeiss) and left undisturbed for 1 h, to allow deposition of the fluorescent particles on the hydrogel surface.

Three different types of spherical indenters were used during the measurement of elastic modulus using the indentation method. For 10 mg/mL samples, a 1/32″ silicon nitride (Si_3_N_4_) indenter was carefully positioned on the hydrogel surface. For 20 and 30 mg/mL, 1/32″ zirconium dioxide (ZrO_2_) and 1/8″ tungsten carbide (WC) were used, respectively. The indentation depth created by the indenters was verified by collecting Z-stack images with a layer thickness of 1 μm and with a refractive index of 1.33. This refractive index was used since indenters were immersed in the water supernatant from the polystyrene solution. Images were obtained on the LSM using a 10× objective lens (EC Plan-Neofluar 10×/0.30 M27) and two excitation sources (488 and 543 nm). An argon laser with a wavelength of 488 nm was used to excited fluorescein salt molecules embedded in the fibrin samples (emission of 495 to 540 nm), while a 543 nm Helium-Neon laser was used to excite red fluorescent polystyrene particles (emission 555 to 700 nm). The indentation depth (δ) created by the indenter in the surface of fibrin gels was measured by visualizing the cross-section images (XZ and YZ) of the Z-stack layers using Zen 2.3 software. Indentation experiments were performed in triplicates and at room temperature (25 ± 2 °C).

Since the determination of the indentation depth is dependent of a flat hydrogel surface, indentation experiments were performed in the center of the hydrogel samples on well plates. Preparation of hydrogel samples on 24-well plates is crucial in order to decrease concavity of the hydrogel samples at the center of the well. In addition, the deposition of the fluorescenet polystyrene particles on the surface of the fibrin samples allowed the verification of a flat hydrogel surface by LSM.

The height of the hydrogel samples was also determined by measurement of Z-stack layers with a thickness of 5 μm using the same excitation sources as described above. The determination of the hydrogel height was performed by calculating the derivative of the fluorescence intensity profile for each channel (green–488 nm and red–543 nm) as described previously [11].

The elastic modulus *E_modified Hertz_* of the fibrin samples were obtained based on the modified Hertz model equations for thin films [12]:

(1) $$\frac{E_{modified\;Hertz}}{E_{Hertz}}=\frac{1+2.3\omega }{1+1.15\omega^{1/3}+\alpha \left(\frac{R}{h}\right)\omega +\beta \left(\frac{R}{h}\right)\omega^{2}}.$$

In which *E_Hertz_* is the value of elastic modulus obtained using the Hertz Theory and *w* is the parameter that correlates the radius of the indenter *R*, with indentation depth δ and sample height *h*.

(2) $$E_{Hertz}=\frac{3\left(1-\nu^{2}\right)F}{4R^{1/2}\delta^{2/3}},$$

(3) ω≡(Rδh2)12.

Considering that hydrogel samples are mainly composed of water, a frictionless condition can be considered between the spherical indenter and the hydrogel surface:

(4) $$\alpha \left(\frac{R}{h}\right)=10.05-0.63\sqrt{\frac{h}{R}}\left(3.1+\frac{h^{2}}{R^{2}}\right),$$

(5) $$\beta \left(\frac{R}{h}\right)=4.8-4.23\left(\frac{h^{2}}{R^{2}}\right).$$

In order to obtain elastic modulus using this modified method, parameters such as indentation depth (*δ*), hydrogel thickness (*h*) and indenter radius (*R*) should be in the range of δ/*h* ≤ min (0.6, *R*/*h*) and 0.3 ≤ *R*/*h* ≤ 12.7 [11].

**Scheme 1:**
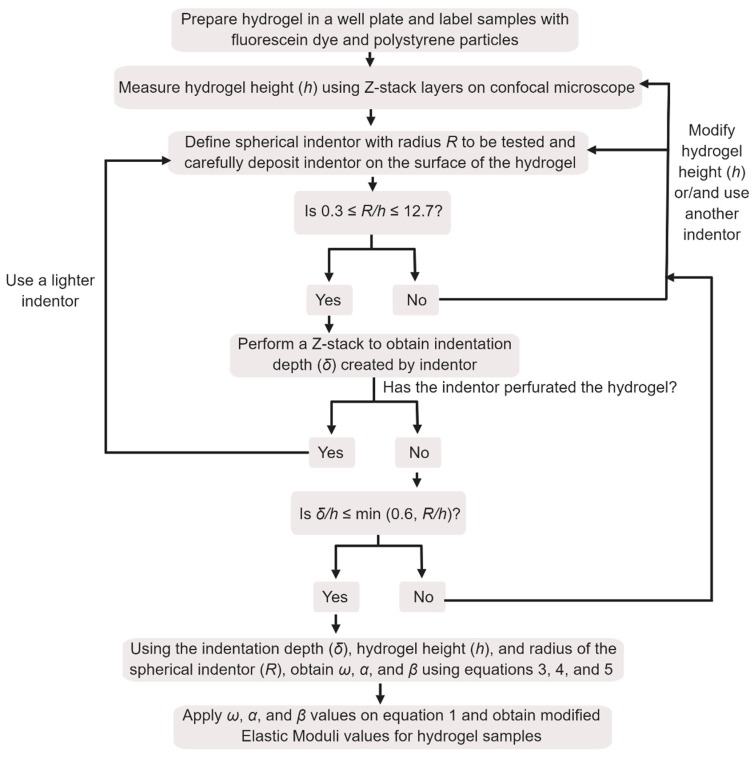
Flow chart for determination of elastic modulus using the indentation method.

Scheme 1 illustrates the steps of the indentation method, from experimental procedure to determination of elastic modulus.

### 2.7. Design and Fabrication of a High-Throughput Electrophysiology System

Adult human motor neurons were cultured on a 96-well plate for seven days to allow for the development of neurite extensions. As a control, hiPSC-derived neural precursors were grown in the same way and tested in parallel. For testing, cells were incubated in FLIPR Blue dye and read by a microplate reader set to excite fluorescence at 530 nm and read fluorescent emission at 565 nm, taking 25 reads from each well in a 5 × 5 grid. Wells containing only FLIPR Blue dye were used to determine background fluorescence (*F˳*). The normalized change in fluorescence, *F−F˳*/*F˳*, was calculated for each reading using the average for *F˳*, and converted to change in voltage membrane potential, ΔE, using the equation below:

(6) $$\Delta E=\frac{R\times T}{z^{\prime }\times F}\times \ln\left(\frac{1}{\frac{\Delta F}{F˳}+1}\right).$$

This equation was previously derived from the Nernst and Goldman–Hodgkin–Katz equations by Fairless et al. who then validated it using patch clamp technique for use with FLIPR Blue dye and neuronal cultures [19]. In this equation, *R* is the gas constant, *F* is Faraday’s constant, and *T* is absolute temperature (*RT/F* = 25.43 mV at 22 °C). *z*′ represents the apparent charge of the external dye concentration and can be interpreted as an experimentally-determined indicator of the sensitivity of the dye. The value of *z*′ is specific to different cell types, and was experimentally determined by Fairless et al. to be between −0.62 and −0.72 for neuronal cultures depending on the neuronal subtype. In our experiments a *z*′ value of −0.64 was assumed. Experimental determination of *z*′ can be found by each user by altering cell membrane potential through the addition of increasing KCl concentrations in the media (5.4, 10, 15, 20, 25 mM for instance) and imaging the resulting fluorescence increase using a fluorescent microscope equipped with a camera. ImageJ, a free open platform for scientific image analysis, can then be used measure pixel intensity changes from the images in areas of interest (single neurons) to determine values for ΔF/F˳ (see Supplemental Information for details). Plotting 1/(ΔF/F˳+1) vs. Kx/Kr (Kx = external K^+^ concentration, Kr = reference concentration 5.4 mM), then gives the value of *z*′ as the slope of the best fit line. For an example of this technique see the study by Fairless et al. [18]. Finally, the coefficient of variation (COV) within each well was calculated and found to be less than 10% overall, with 85% being less than 5%, verifying that this average serves as a good representation of the overall resting activity of each well. We tested cholinergic neurons (spinal cord motor neurons) using acetycholine (100 µM) as a stimulant, however if the neuronal subtype of the culture is unknown or the population is mixed, potassium chloride (56 mM) is a general stimulant that can be used. Some plate readers require that the plate lid be removed, so to maintain sterility of the neural cultures, plate lids were replaced before readings with optically clear adhesive plate seals under sterile conditions.

## 3. Results

### 3.1. Validation of an Indentation Protocol for Directly Measuring the Elastic Modulus of Hydrogels

**Figure 1:**
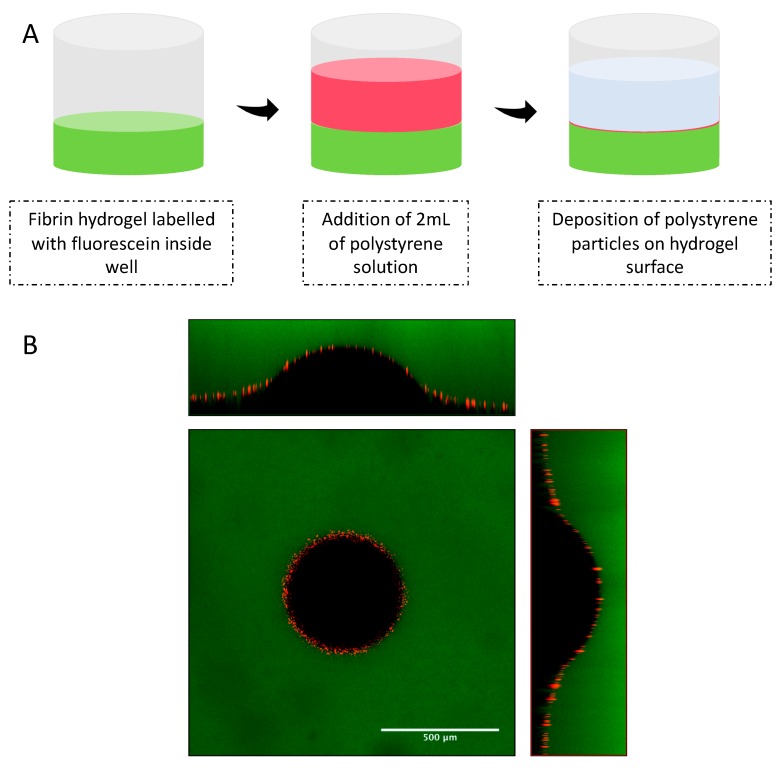
Set-up for indentation experiment: (**a**) Hydrogel samples were labelled with fluorescein sodium salt (green) while red polystyrene particles were deposited on its surface (red). Spherical ceramic indenters were positioned on the surface of the fibrin samples, generating an indentation depth. (**b**) Indentation depths were measured by visualizing cross section images obtained in the axis of symmetry of the gel indented surface. Scale bar represents 500 μm.

Elastic modulus was evaluated for hydrogel samples prepared with fibrin concentrations of 10, 20 and 30 mg/ml. Hydrogel samples were stained with fluorescein sodium salt in order to obtain a more precise measurement of hydrogel thickness. In addition, deposition of red fluorescent polystyrene particles on the surface of the samples allowed a better visualization of indentation depth created on the hydrogels. The indentation depth caused by the mass of the indenter was determined by XZ- and YZ-cross section images of the hydrogel samples. Figure 1 displays the indentation depth profile obtained during the indentation experiment.

**Figure 2:**
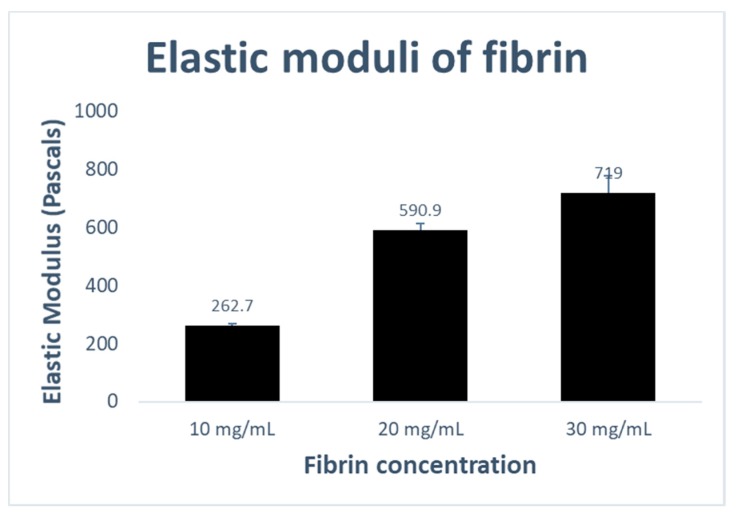
Elastic moduli values obtained from modified Hertz model for thin films. Data is presented as the average and standard deviation of three sample replicates.

Measurements of elastic moduli of 10 mg/mL fibrin samples were performed using a 1/32″ silicon nitride (density of 3.184 g/cm^3^) spherical indenter. Figure 2 displays elastic moduli values obtained for these samples. Indentation experiments performed in 20 mg/mL samples previously used the same indenter defined for 10 mg/mL samples. However, since the indentation depth created by the 1/32″ silicon nitride indenter on the surface of the 20 mg/mL fibrin samples was not considerable, a denser indenter was selected (1/32″ zirconium dioxide with density of 5.680 g/cm^3^). As shown in Table 1, 20 mg/mL fibrin samples with 1/32″ zirconium dioxide indenter generated a R/h of 0.3 ± 0.002.

In the case of samples with concentrations of 30 mg/mL, a heavier indenter of 1/8″ tungsten carbide (density of 15.630 g/cm^3^) indenter was defined due to the high elastic modulus of this sample. As expected, with increase of fibrin concentration, elastic moduli values also increased.

### 3.2. Validation of a High-Throughput Electrophysiology System

**Figure 3:**
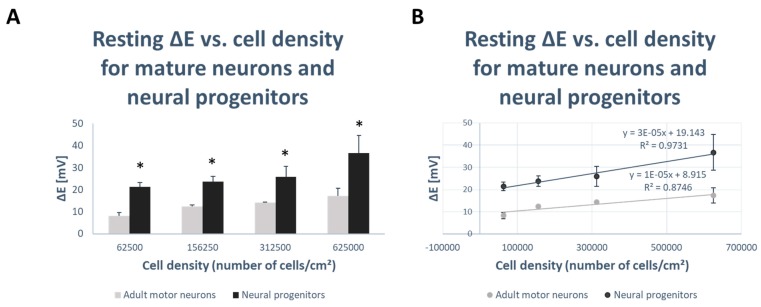
Microplate readings of mature and immature resting neural cultures. (**A**) The resting membrane potential of mature neurons versus immature neural progenitors at varying cell densities shows that the more hyperpolarized membranes of mature neurons are detectable using our method. (**B**) The relationship between the resting membrane potential readings and cell densities is linear with a high correlation coefficient of 0.97. Data is presented as the average and standard deviation of three biological replicates. Statistics were performed by one-way student *t*-test assuming two sample equal variance. * signifies statistical significance between the Neural progenitor readings versus the Adult motor neuron readings.

Mature human motor neuron cultures were tested in parallel with immature neural precursor cultures to validate the ability of our protocol to detect electrophysical maturation in neuronal cultures. Figure 3A shows that the motor neuron population possessed a significantly more hyperpolarized resting membrane potential than the immature neuron population, as expected, illustrating the ability of this protocol to reliably differentiate between electrophysically mature and immature resting membrane potentials in neuronal cultures. Furthermore, our protocol was consistent regardless of cell culture density. These values were then correlated with varying cell culture densities to note the relationship between the overall fluorescence with number of cells, since in a homogenous population such as this the relationship should be linear if the microplate is reading the dye reliably. Figure 3B shows that the relationship between ΔE and the cell density of the neural cultures is linear with a high correlation coefficient of 0.97 for the immature neurons, and 0.87 for the motor neurons, indicating that the microplate reader is indeed reading the dye in a reliable fashion.

**Figure 4:**
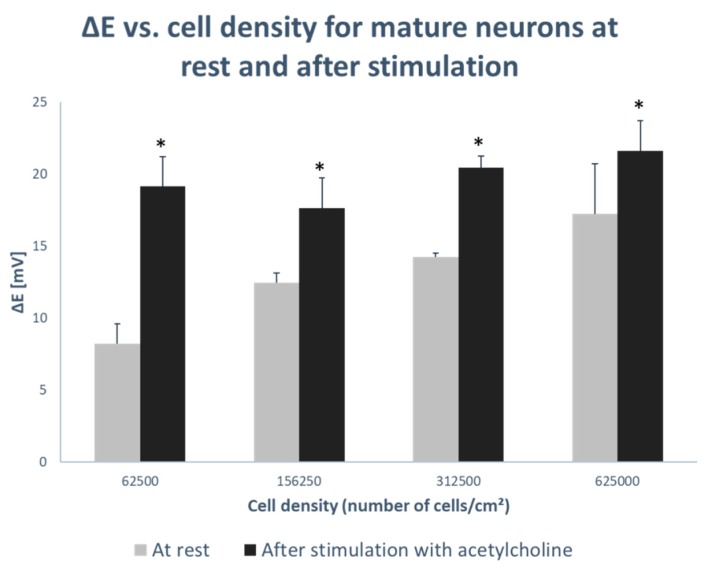
Microplate readings of mature neurons before and after stimulation. Data is presented as the average and standard deviation of three biological replicates. Statistics were performed by one-way paired student *t*-test. * signifies statistical significance between the After stimulation with acetylcholine readings versus the At rest readings.

Lastly, the cells were stimulated by incubation in the neurotransmitter acetylcholine for 15 min and read in the microplate reader again. The readings from each well were then averaged and compared to the previous readings of the cells before stimulation as seen in Figure 4. It was noted that readings within each well after stimulation had a COV of less than 5% (*n* = 25), and the averages for each well replicate (*n* = 3) were also in close agreement with one another. These readings were also significantly higher than the readings taken prior to stimulation in each case, providing evidence that this protocol can reliably detect electrophysical stimulation in neuronal cultures. Finally, the activity of the cells loaded with dye was confirmed visually using a microscope equipped with fluorescence. At an exposure of 250ms and maximum gain, electrophysically active cells could be seen to increase in intensity quite clearly (see Supplemental image stack Figure S1). 

## 4. Discussion

In this paper, we have optimized and validated a set of tools for determination of the elastic moduli and electrophysical maturity of lab-generated spinal cord tissues. When growing neural tissues, it is important that elastic modulus of the scaffolding is soft (0.1–0.5 kPa) to promote neuronal differentiation from neural precursor cell populations [8,9]. It is highly important to generate efficient yields of neuronal cells, as neural precursors favor proliferation over differentiation at higher elastic moduli (3.5 kPa) [7]. With the emergence of 3D bioprinting the challenge of defining scaffolds for neural tissues has become even more complex, as bioprinting requires additional mechanical properties such as rapid crosslinking speeds. Fibrin, a natural hydrogel, is widely reported to support neural differentiation from stem cells and has been used as bioinks to produce 3D bioprinted neural tissues [27,28,29,30,31,32,33,34]. Typical formulations of fibrin in these reports ranges from 10 to 20 mg/mL so we hypothesized that their elastic moduli would fall within the 100–500 Pa range accordingly. We tested 10, 20 and 30 mg/mL fibrin and found their elastic moduli to be 262.7 ± 5.7, 590.9 ± 23.5 and 719.0 ± 58.3 Pa, respectively, as expected.

Standard elastic modulus methods such as AFM and rheology are challenging techniques to apply to hydrogel samples, and this contributes to errors and uncertainties in measurements. In the case of AFM measurements, selection of cantilevers with appropriate spring constant values that will allow approach and retraction movements without damaging the hydrogel samples is time-consuming and expensive. Rheological measurements of soft hydrogel samples are also challenging, considering that it is very easy to damage the sample by compression. In addition, elastic modulus is obtained indirectly, from a relationship between shear and elastic moduli.

The indentation method allows the determination of elastic modulus of hydrogel samples by combining spherical hydrogels and fluorescence microscopy. As shown in Table 1, for ultrasoft samples (10–20 mg/mL), the elastic moduli values obtained with the modified model do not differ considerably from the values obtained with the Hertz Theory. However, for stiffer hydrogel samples, such as 30 mg/mL fibrin, the Hertz Theory displays very high elastic moduli values when compared to values experimentally determined. These results emphasize the necessity of proper models to define elastic moduli for hydrogel samples.

Just as the elastic modulus of the scaffolding is important to supporting the generation of neural tissues, the presence of electrophysical signaling is equally as important to support their function as transmitters of information. We described a system to measure membrane potential in neural cultures using the voltage sensitive dye FLIPR Blue. Additionally, by adding ion channel blockers or neurotransmitters to the media, this system can be used to investigate specific ion channel responses in the cultures [20,21]. We showed that this system can reliably detect statistically significant differences in fluorescence levels between electrophysically immature versus mature neural cultures, as well as stimulated versus resting neural cultures. Altering cell culture densities from very low to very high had no effect on the protocol, illustrating its robustness and suitability for neural tissue analysis where self-division of stem cells during neural differentiation means that final neuronal cell densities cannot be predetermined. Furthermore, each experimental test required less than 10 min to read, allowing for several tests to be performed within the time frame of a single application of the dye.

## 5. Conclusions

Here we have demonstrated a set of direct methods optimized for the first time for measuring the elastic moduli of soft fibrin hydrogels as well as for detecting the electrophysiological character of motor neurons using a voltage sensitive dye. Taken together these tools provide a means to interrogate the mechanical and electrophysical properties of engineered spinal cord tissues without loss of cell viability. Using these tools to acquire direct measurements in living spinal cord tissues will allow researchers to fine-tune the properties of these engineered constructs to ultimately produce smarter regenerative strategies for spinal cord injury.

## Figures and Tables

**Table 1:** Indentation parameters and elastic moduli values obtained for fibrin samples.

Indentation Results	10 mg/mL	20 mg/mL	30 mg/mL
0.3 ≤ R/h ≤ 12.7	0.33 ± 0.03	0.30 ± 0.002	1.24 ± 0.03
δ/h ≤ min (0.6, R/h)	0.05 ± 0.005	0.04 ± 0.001	0.48 ± 0.02
E_Hertz_	299.6 ± 9.7	657.1 ± 25.9	2260.9 ± 186.8
E_modified Hertz_	262.7 ± 5.7	590.9 ± 23.5	719.0 ± 58.3

## Supplementary Materials

The following are available online at https://www.mdpi.com/2079-6374/9/2/51/s1, Figure S1: stack image set of active motor neuron culture visualized with Fluorescence Imaging Plate Reader (FLIPR) Blue dye: time-lapse images taken every 500 ms show the intensity differentials of the dye as neurons fire spontaneously. The method for determining fluorescence intensity values from images using ImageJ, and the ImageJ software are available https://theolb.readthedocs.io/en/latest/imaging/measuring-cell-fluorescence-using-imagej.html.
